# The yield of HIV testing during pregnancy and postnatal period, Uganda, 2015–2018: analysis of surveillance data

**DOI:** 10.1186/s12981-021-00360-0

**Published:** 2021-06-24

**Authors:** Yvette Wibabara, Ivan Lukabwe, Irene Kyamwine, Benon Kwesiga, Alex R. Ario, Linda Nabitaka, Lilian Bulage, Julie Harris, Peter Mudiope

**Affiliations:** 1 Uganda Public Health Fellowship Program, Ministry of Health, Kampala, Uganda; 2 Sexually Transmitted Infections/AIDS Control Program, Ministry of Health, Kampala, Uganda; 3 United States Centers for Disease Control and Prevention, Kampala, Uganda

**Keywords:** HIV testing, Yield, Pregnancy, Post-natal, Uganda

## Abstract

**Background:**

Uganda has registered a reduction in new HIV infections among children in recent years. However, mother-to-child transmission of HIV still occurs, especially among pregnant women who present late. To eliminate this transmission, all HIV-positive pregnant women should be identified during antenatal HIV testing. We described women newly identified HIV-positive during pregnancy and postnatal period 2015–2018.

**Methods:**

We extracted surveillance data for women identified as HIV-positive during pregnancy and the postnatal period reported through the Health Management Information System from 2015–2018. We calculated proportions newly positive at antenatal, labor, and postnatal periods nationally and at district levels. We disaggregated data into ‘tested early’ (during antenatal care) and ‘tested late’ (during labor or postnatal period) and calculated the proportion positive. We evaluated trends in these parameters at national and district levels.

**Results:**

Overall, 8,485,854 mothers were tested for HIV during this period. Of these, 2.4% tested HIV-positive for the first time. While the total number of mothers tested increased from 1,327,022 in 2015 to 2,514,212 in 2018, the proportion testing HIV-positive decreased from 3.0% in 2015 to 1.7% in 2018 (43% decline over the study period, p < 0.001). Of 6,781,047 tested early, 2.2% tested HIV-positive. The proportion positive among those tested early dropped from 2.5% in 2015 to 1.7% in 2018. Of 1,704,807 tested late, 3.2% tested HIV-positive. The proportion positive among those tested late dropped from 5.2% in 2015 to 1.6% in 2018. At the district level, Kalangala District had the highest proportion testing positive at 13% (909/11,312) in 2015; this dropped to 5.2% (169/3278) in 2018.

**Conclusion:**

The proportion of women newly testing HIV-positive during pregnancy and postnatal declined significantly during 2015–2018. A higher proportion of mothers who tested late vs early were HIV-positive. Failure to identify HIV early represents an increased risk of transmission. Ministry of Health should strengthen Elimination of Mother to Child Transmission (eMTCT) services to sustain this decrease through targeted interventions for poorly-performing districts. It should strengthen community-based health education on antenatal care and HIV testing and enhance the implementation of other primary prevention strategies targeting adolescents and young women.

## Background

Globally, approximately 90% of children with HIV infection are infected during gestation, delivery, or breastfeeding [1]. In situations where infection control practices adhere to standard precautions and safe blood transfusion it available, mother-to-child transmission of HIV (MTCT) is virtually the only way that infants acquire HIV. Without any interventions for prevention of mother-to-child transmission (PMTCT), the risk of an infant acquiring HIV infection from an HIV-positive mother in developing countries is 25–45% [2]. Despite marked progress in reducing the new HIV infections in Uganda, particularly among children, and minimizing AIDS-related deaths, the country continues to have a high burden of HIV. The most recent Uganda Population-Based HIV Impact Assessment (UPHIA), conducted in 2016–2017, demonstrated a prevalence of 6.2% overall among persons 15–64 years of age. The prevalence was even higher among women, at 7.6%, compared to 4.7% among men [3].

In Uganda, since the early 1980s, MTCT has been the second most-common mode of HIV infection, accounting for about 18% of new infections [4]. However, since implementing PMTCT interventions in 2012, there has been a dramatic reduction in new vertical infections from 25,000 in the year 2000 to approximately 2,300 in 2019 [5]. PMTCT is a four-pronged approach comprising of a package of interventions summarized in areas: 1) primary prevention of HIV infection; 2) prevention of unintended pregnancies among women living with HIV; 3) prevention of HIV transmission from women living with HIV to their infants; and 4) provision of treatment, care, and support to women infected with HIV, their children and their families. This is part of the elimination of mother-to-child-transmission strategy recommended in the HIV treatment guidelines [5]. In addition, there has been an increase in the proportion of women tested during pregnancy in Uganda, with 98% of pregnant women who attended ANC in 2019 knowing their HIV status [6].

Antiretroviral Therapy (ART) provision to the public and private sector in Uganda has also increased. This has been accelerated by the test and treat policy was adopted in Uganda on December 1, 2016 [3].

Despite these interventions, MTCT still occurs, primarily among women who present late for care during pregnancy as well as some who are missed during pregnancy [6]. This hinders achievement of the elimination of mother-to-child transmission (eMTCT) target of reaching MTCT rates of < 5% by 2021. Results from a systematic review suggest that women living in regions where HIV infection is common are at high risk of acquiring HIV infection during pregnancy and the postpartum period, and that mothers who acquire HIV during pregnancy or postpartum are more likely to pass the infection to their infants than mothers with chronic HIV infection [7]. In order to successfully eliminate vertical transmission, all HIV-positive women need to be identified through routine antenatal care (ANC) HIV testing at all stages of pregnancy as well as through the post-natal and breastfeeding periods [5]. We described HIV-positive mothers identified during pregnancy and post-natal periods using Health Management Information System (HMIS) data from 2015–2018. Findings from this analysis will guide stakeholders on developing targeted eMTCT interventions in Uganda.

## Methods

### Study design and data source

We conducted secondary analysis of routinely-collected program surveillance data on HIV testing for women who attended first ANC, delivered in a health facility, or attended postnatal care (PNC) from 2015–2018 in Uganda. HIV routine testing is by rapid antibody-based tests. We extracted data from the electronic District Health Information System version 2 (DHIS2). The HMIS is an integrated reporting system that includes: Monthly attendance numbers from the outpatient department (OPD), OPD diagnoses, Maternal and Child Health Services (MCH), HIV/AIDS service data, Laboratory data, stock outs of essential drugs and supplies, and financial data. The section on MCH includes sub-sections on ANC, Labor/Delivery and Post Natal. This information is loaded on an electronic web-based system called the DHIS2 [8].

### Study population

All Ugandan women in all districts in Uganda that attended first ANC, delivered in a health facility or attended the PNC from 2015–2018 as recorded in the DHIS2.

### Study variables and data abstraction

We extracted data from the DHIS2 (specifically HMIS Form 105, which is the Health Unit Outpatient Monthly Report). We extracted data on the following variables: newly tested for HIV during pregnancy, tested HIV-positive for the first time during pregnancy, re-tested later in pregnancy, tested HIV-positive on a retest, total tested for HIV in labor (first time testing during this pregnancy), tested HIV-positive in labor (first time testing during this pregnancy), tested for HIV in labor (Retest this Pregnancy), tested HIV-positive in labor (retest this pregnancy), breastfeeding mothers tested for HIV (first test), breastfeeding mothers newly testing HIV-positive (first test), breastfeeding mothers tested for HIV (retest), and breastfeeding mothers newly testing HIV-positive (retest).

### Data analysis

The data for each of the districts were extracted from DHIS2 and exported to Microsoft Excel and EpiInfo for analysis. We calculated proportions of women that tested positive at ANC, Labor, and PNC at national and regional levels. We categorized women into those who tested early (during ANC) and tested late (during labor/delivery and PNC). We calculated the HIV yield among those testing early and late at national and district levels. HIV yield was defined as the proportion of women who newly tested HIV-positive out of the total women tested for HIV (Total newly Testing HIV-positive/ Total number of women tested for HIV). We used line graphs to demonstrate the national trend and maps to demonstrate the trend at district level. We used logistic regression to determine the statistical significance of the trend.

## Results

### HIV testing yield during pregnancy and postnatal period, Uganda, 2015–2018

A total of 8,485,854 pregnant women were newly tested during ANC, labor/delivery, and breastfeeding periods during 2015–2018. Of these, 200,786 (2.4%) tested HIV-positive. A total of 6,781,047 tested early with 146,012 (2.2%) testing positive, while 1,704,807 tested late with a yield of 54,774 (3.2%) (Table 1). The overall HIV yield decreased from 3.0% in 2015 to 1.7% in 2018 (43% decrease, p < 0.001) (χ^2^ for trend). The yield among those tested early dropped from 2.5% in 2015 to 1.7% in 2018, while that among those tested late decreased from 5.2% in 2015 to 1.6% in 2018 (Table 1). The yield between those tested early and those tested late was statistically different (p < 0.001).

**Table 1:** Proportion of newly tested HIV positive in antenatal (early), labor and postnatal period (late), Uganda, 2015–2018

Year	Number tested early	Number tested late	Tested early % positive	Tested late % positive	P value
2015	1,061,165	265,857	2.5	5.2	< 0.0001
2016	1,839,948	431,112	2.6	4.3	< 0.0001
2017	1,879,385	494,175	2.1	2.9	< 0.0001
2018	2,000,549	513,663	1.7	1.6	< 0.0001
Total	6,781,047	1,704,807	2.2	3.2	< 0.0001

**Fig. 1:**
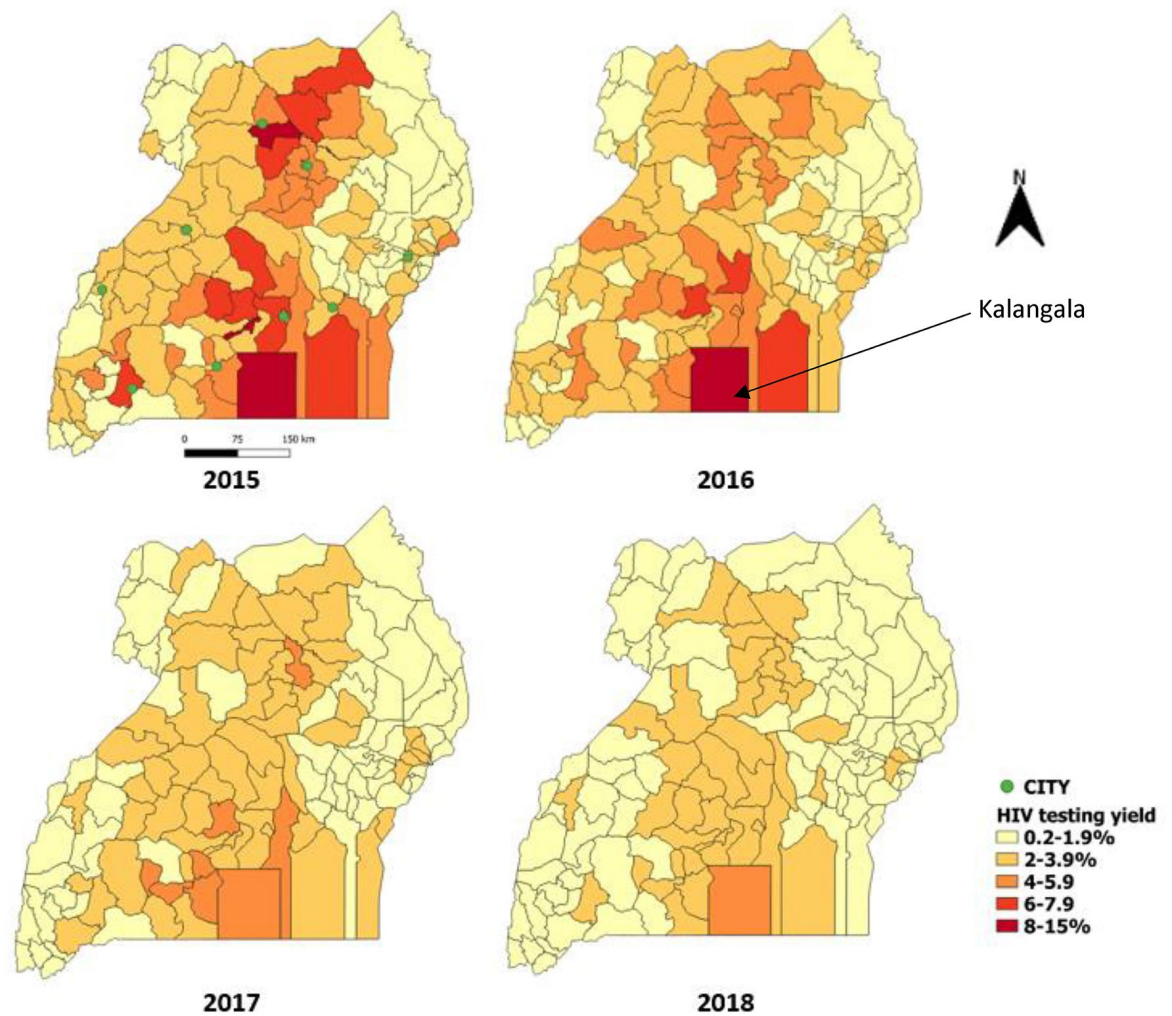
Overall HIV testing yield during pregnancy and postnatal period by district, Uganda, 2015–2018

**Fig. 2:**
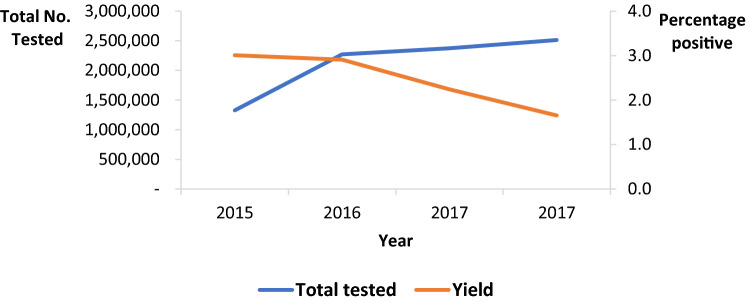
Trend of the HIV testing yield and Total number of tests done during pregnancy and postnatal periods, Uganda 2015–2018

The decrease was more pronounced in the mid-north and the central regions than in other regions of the country. Kalangala District had a persistent higher yield over the years, compared with all the other districts. Nevertheless, it also recorded a decrease from 13% in 2015 to 5.2% in 2018 (Fig. 1). During this period, the total numbers of mothers tested approximately doubled (Fig. 2).

**Fig. 3:**
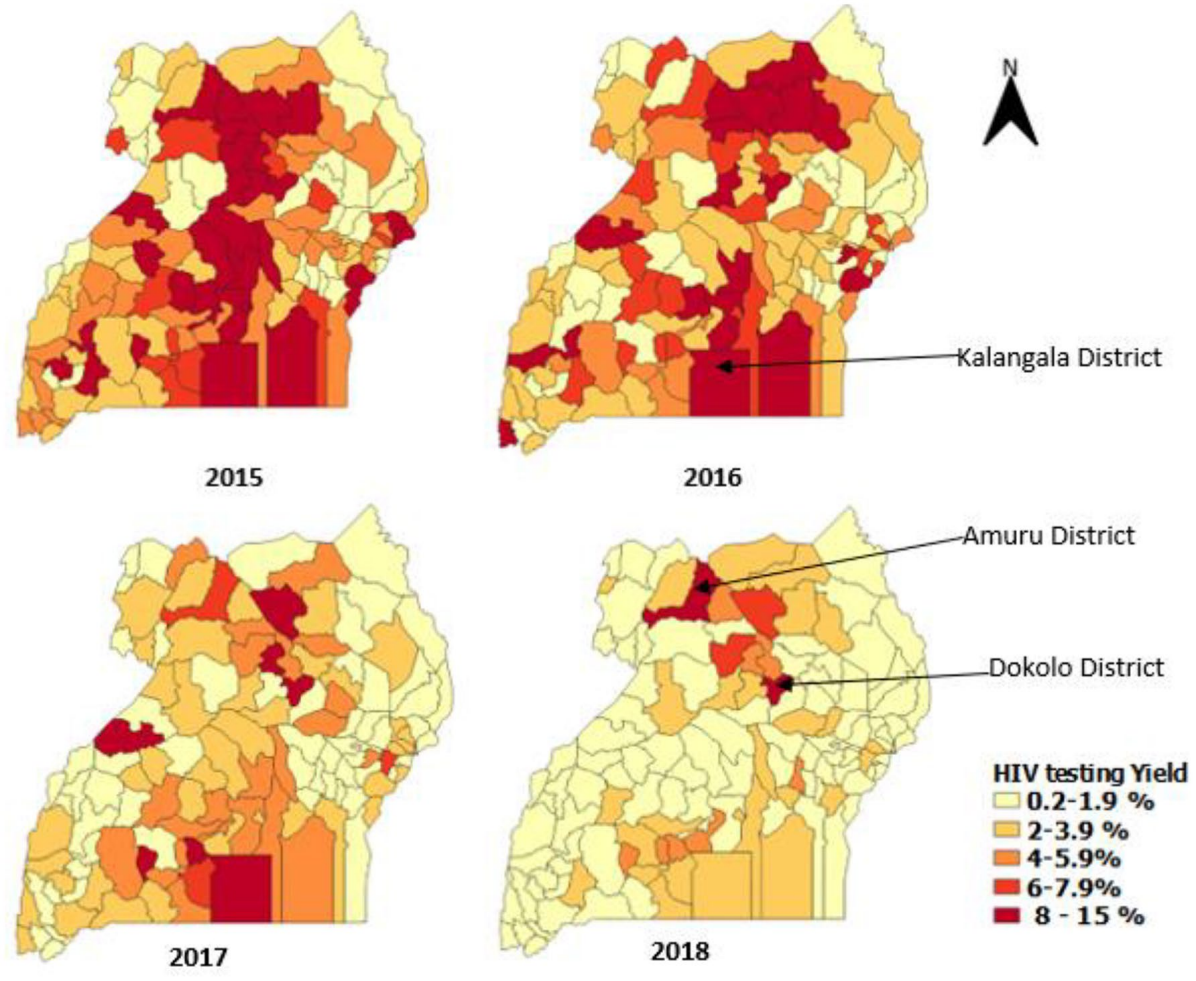
HIV testing positivity among mothers tested late (labor/delivery and post-natal period), by district, Uganda, 2015–2018

**Fig. 4:**
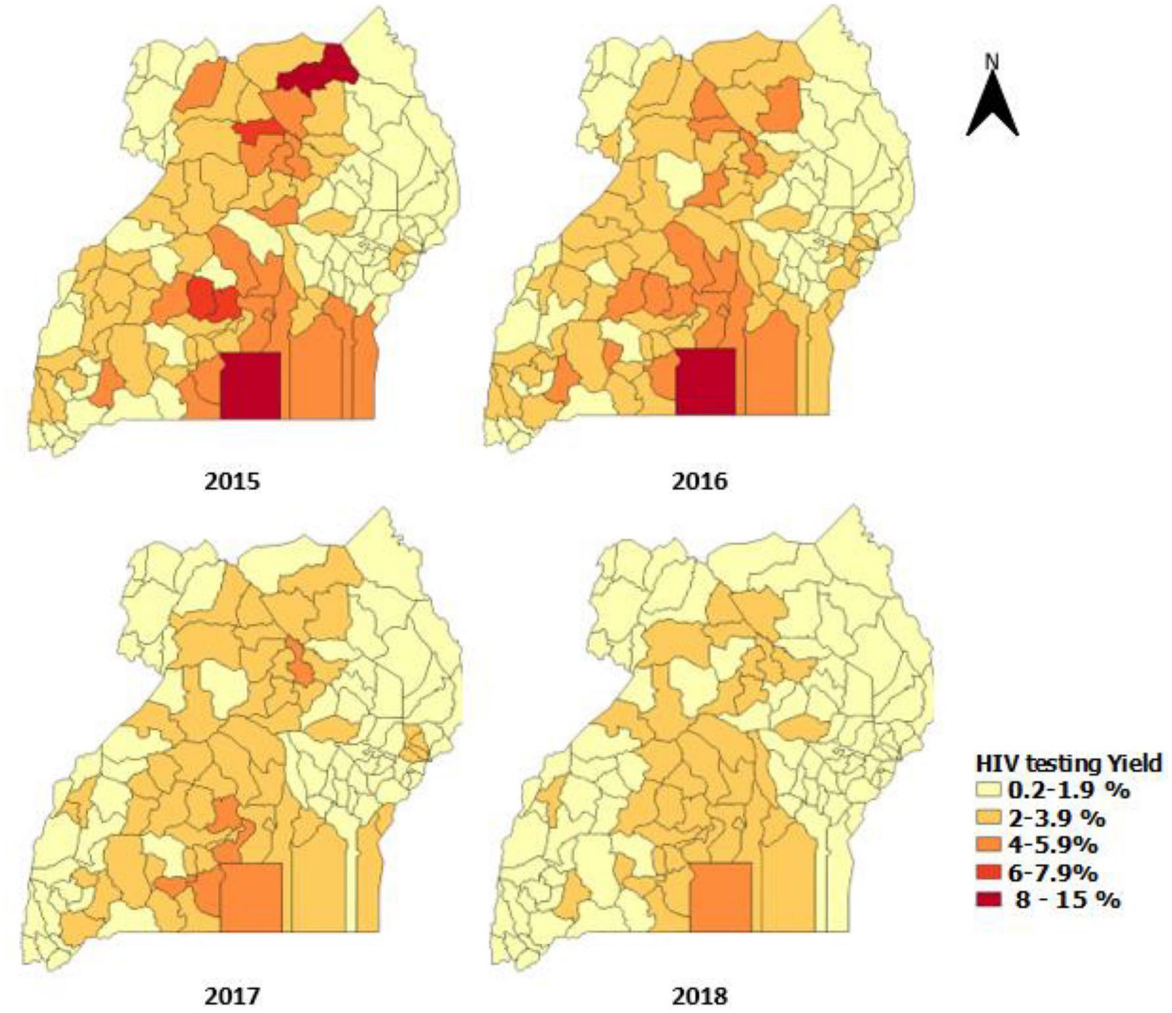
HIV testing positivity among mothers tested early during antenatal, by district, Uganda, 2015–2018

When comparing the trends in HIV yield between those who tested early and those who tested late, there was a general decrease in the yield, but it was more pronounced among those who tested late (5.2% in 2015 to 1.6% in 2018) (Fig. 3). Among those that tested late, most of the districts recorded a yield above 8% in 2015 and 2016. This was mainly in the mid-north, central, and a few districts in the east, west and south western parts of Uganda. In 2018, the yield was highest in mid-north, especially the districts of Amuru, and Dokolo at 9.1% and part of central region (Kalangala District) (Fig. 4). The central region persistently had a higher yield compared with other regions, most pronounced in Kalangala District (Fig. 4).

## Discussion

There was a decrease in the proportion of women testing HIV-positive during the pregnancy and breastfeeding periods in Uganda during 2015–2018, along with a coincident increase in testing among women. Declines in HIV testing yield (proportion positive out of all tested) were observed among women testing early and women testing late, but were more pronounced in women testing late. This underscores the success of several programs designed to diagnose and treat persons living with HIV in Uganda, including pregnant women.

The decrease may be at least partially attributable to Uganda’s PMTCT program, especially Prong Three, which focuses on improving utilization of Maternal Child Health (MCH)/PMTCT services among HIV-positive pregnant mothers. Although the main focus of the program is to prevent MTCT, the program also has components aimed at keeping women who test HIV-negative free of HIV infection, including counselling and support, male partner involvement, and condom use [5]. Policy makers need to ensure that strategies and activities addressing the first two prongs of PMTCT are included (with appropriate resource allocations) in the development and implementation of national plans and strategies for the elimination of pediatric HIV [9].

While the yield of HIV-infected persons per test decreased, the total number of HIV tests performed increased. The increase is likely due in part to the roll-out of the HIV Testing Services (HTS) policy and implementation guidelines, which were enacted in Uganda in 2016 and were aimed at increasing the number of persons that test for HIV. The HTS policy emphasizes that HIV testing should be done as early as possible during pregnancy to enable pregnant women with HIV to obtain and benefit most from prevention services offered [10]. The decrease in yield with an increase in number of tests done provides support for a true decline in prevalence, which underscores the success of the program. Of the nearly 2 million women attending their first antenatal clinic services in 2018, of whom 6% were infected with HIV, only 1.8% were learning their status for the first time [11].

Despite national progress, specific districts still appear to face challenges and require targeted interventions. In particular, the northern region of Uganda had a smaller decline in testing yield, compared to other regions. These regions have had less success in the implementation of various HIV-related interventions, as measured by health indicators in the Health Performance reports from 2015 to 2019 [12]. In addition, these districts are also known to have less robust health-seeking behavior in their population compared to other regions [13]. Other districts, such as Kalangala District, may have special needs. As an island district with challenging geography and difficult access, faces challenges across multiple health issue [14]. Specific, targeted efforts may be required to achieve the desired results in these districts.

Much as HIV testing should be done as early as possible during pregnancy to reduce the risk of HIV transmission to infants, it is evident from this study that there is still a proportion of women who present late to health facilities for antenatal care. The highest proportion of late testers was in Mid-Northern and part of Central region of Uganda (Kalangala district) in 2015, 2016, and 2017. Previous studies in Uganda have shown late ANC presentation to be associated with absence of complications and a perceived lack of need for ANC, long distance to the facility and/or challenging terrain, personal discomfort with seeking ANC, and others [15, 16]. Indeed, women may not be aware of the importance of ANC care, particularly in light of an uncomplicated pregnancy. In a review article of barriers to antenatal care seeking in women in Kenya, Rwanda and Nigeria, barriers to fully utilizing ANC and delivery services included the fear of testing positive for HIV, attitudes of healthcare providers, long clinic waiting time, and costs of both services delivered and transport and access to the health facility [16]. Further research is needed to identify optimal messaging and modes of communication to improve early presentation for ANC in Uganda.

## Limitations

Because DHIS2 data are aggregated and do not represent patient-level data, we could not describe the outcomes in more granular terms. Individual characteristics associated with high testing yield could not be explored. There may also be selection biases that reduce data generalizability, due to the distribution and utilization of public and private ANC services.

## Conclusion

The proportion of mothers newly testing HIV positive during pregnancy and post-natal period declined from 2015–2018, which likely reflects the impact of PMTCT interventions and Uganda’s overall HIV epidemic control strategy. The infection prevalence was highest among pregnant women testing late, which reflects a period during which risk of MTCT is highest. The MoH should strengthen eMTCT services to sustain this decrease, as well as design targeted interventions such as community-based health education on the importance of early antenatal care and timely HIV testing during pregnancy for districts with high infection prevalence.

## Data Availability

The data upon which our findings are based belongs to the government of Uganda, Ministry of Health and cannot be shared publicly. However, the data can be availed by the corresponding author with permission from the Ministry of Health Uganda Division of Health Information.

## Abbreviations

ANC: Antenatal care
DHIS2: District Health Information System version 2
eMTCT: Elimination of mother-to-child HIV transmission
HIV: Human immunodeficiency virus
HMIS: Health management information system
HTS: HIV testing services
MCH: Maternal child health
MNCAH: Maternal, newborn, child and adolescent health
MOH: Ministry of health
NPAP: National Priority Action Plan for HIV/AIDS
OPD: Outpatient department
PNC: Postnatal care
SMC: Safe male circumcision
UPHIA: Uganda population-based HIV impact assessment
